# Without Teeth

**Published:** 1858-08

**Authors:** 


August 14th, 1858.

DEAR FRIEND:

In accordance with your request I have chipped out a piece of my brain ; I have made it a little spicy—it is necessary. However, you will find almost any sentiment from love to the devil in it. It is, or has been, hastily composed. I had to get up on top of the chimney two or times to catch an inspiration.

The people in the vicinage would have thought me partially deranged if they had not seen me sky-larking before. The only damage it has done is to the roof, it may leak a little, but that's of no consequence so long as the weather remains dry.

### WITHOUT TEETH,

"The way of Life is Death."

BY A. S. P.

I was charmed with a lassie, whose face was very fair,
As the laughing waters, moon-lit, flowed her flaxen hair,
And waved those golden ringlets, around her eyes of night,
Those eyes that were as mirrors, where bathes the moon's first light,
With a tinge of roses under, that glows about the sun,
I saw! I gazed! admired, my heart was fairly won.

Her lips they were as cherries red, that tempt the youthful hand,
I thought to taste such nectar, were to be in fairy land;
Then softly stealing close to her, I sat down by her side,
Her eyes were full of beauty, as they seemed my sloth to chide;
They beamed so very merry as they laughed above those lips,
That my hand unconscious sought and clasped her finger tips.

I asked a drop of nectar, a drop that should be mine,
At once to taste those ruby lips, to taste her lips divine;
She spoke, her lips were parted, and I thought to see the pearls,
The sun-lit pointed icicles, to match her sunny curls;
She said that I was welcome, when I saw!—how can I tell—
I saw a scraggy pit-fall, that breathed perfumes of h—ll.

Dread visions of the sepulchre, came shadowing o'er my mind,
I thought of demons haunting, my life in forms refined,
As they stole up through the shadows, from out the land of hades,
Wearing glorious sunlight, while their hearts were dark wi' shades;
That their breath was pestilential, breathing chol'ra and plagues,
So fancied that my last resort depended on my legs.

Thus fly the dread contagion, that my being would undo,
Houses, alleys, streets, and pavements, like magic before me flew;
'Till I turned around a corner, when slap upon my back,
Came friendly hand and voice, saying, is death upon your track;
"Worse! worse! than death," the foulest air has almost ta'en my breath,
I flee a main, I flee in pain, to save myself from death.

I said, my friend, come with me, here together let's not wait;
I can't, said he, I'm going to Toland's, at thirty-eight.
What's there, said I? There's diamonds, there's teeth of fairest pearls,
To make beauties of creation, to beautify the girls;
I have orders for Miss M————, you know she is very fair,
They'll make her mouth as brilliant, as her flowing sunny hair

Soothed, I went to Fourth street, to Toland's, in thirty-eight,
To see what could be done for the caverns of the pate;
There were rain drops, strewn and laying in rows together,
To suit all tints, complexions, make foulest breath fair weather;
I asked him if he ventured within those dark abodes,
The sepulchres of life, where foulest breath the health corrodes.

He laughed, and said, most truly, with an instrument of steel,
That I get at number thirty-eight, I work for human weal;
*Forcing up* the snags and roots, so to clear the stumpy ground,
For a crop of gold enamel, that beauty ever crowned;
So tell your friends and neighbors, with Toland for my aid,
I'll set their mouths wi' canny pearls, defy the deeps of hades.

